# Geriatric Assessment as a qualification element for elective and emergency cholecystectomy in older patients

**DOI:** 10.1186/s13017-016-0094-1

**Published:** 2016-07-29

**Authors:** Jakub Kenig, Piotr Wałęga, Urszula Olszewska, Aleksander Konturek, Wojciech Nowak

**Affiliations:** 3rd Department of General Surgery, Jagiellonian University Medical College, Pradnicka str. 35-37, 31-202 Kraków, Poland

**Keywords:** Geriatric assessment, Frailty, Surgery in older patients, Cholecystectomy

## Abstract

**Background:**

Older patients experience a higher incidence of postoperative complications after cholecystectomy compared with younger patients. However, most studies have not considered patient frailty, particularly regarding emergency cholecystectomy. The aim of this prospective study was to evaluate outcomes in frail older patients eligible for elective and emergency cholecystectomy.

**Methods:**

Preoperative Geriatric Assessment (GA) was performed in consecutive patients aged 65+ years, operated for biliary disease. The GA evaluated the functional, cognitive, comorbidity, depressive, nutritional, and polypharmacy status and patients with two or more abnormal domains were considered frail. Outcomes of interest were 30-day postoperative mortality, morbidity, and length of hospital stay (LOS).

**Results:**

A total of 126 patients (median age 74; range 65–93 years) were included. There was no difference between elective frail and non-frail patients regarding postoperative mortality (0 %) and morbidity (6 % vs. 5 %; *p* = 0.76). LOS was not significantly longer in the frail group (5.6 vs. 4 days; *p* = 0.22). In the emergency-admitted patients, almost all complications occurred in the frail population (mortality 5 % vs. 0 %; morbidity 36.7 % vs. 3.3 %, compared with non-frail patients, respectively; *p* < 0.01) and LOS was significantly longer (10.3 (frail) vs. 6 days (non-frail);*p* = 0.03). Frail status was a significant independent predictive factor for postoperative complications in the emergency population, only (odds ratio: 3.4 (1.2–9.7); *p* = 0.02).

**Conclusions:**

Elective laparoscopic cholecystectomy is a safe and effective surgical technique also for older frail patients. In emergency settings, frail patients have significantly more complications and a longer LOS. However, the role of severity of frailty and the most reliable GA tools require further study.

**Trial registration:**

ISRCTN14976998 (retrospectively registered)

## Background

Several studies, concerning surgical treatment of biliary disease, have shown that older patients more often have complicated gallstone disease, more emergency and open operations, higher postoperative morbidity and mortality rates, and a longer postoperative hospital stay compared with younger patients [1–6]. However, most studies have not considered the larger heterogeneity of co-morbidity and physical strength in this group of patients, which further contributes to the complexity of treatment. This relates especially to frail patients who have reduced physiologic reserve associated with increased susceptibility to disability because of age-related loss of physical, cognitive, social, and physiological functioning [7]. In these patients, standard preoperative medical history, physical examination, biochemistry, and imaging tests often do not provide the information needed for optimal and tailored treatment, leading to both under- and overtreatment [8]. On the other hand, a Geriatric Assessment (GA), can provide a comprehensive health appraisal to guide targeted geriatric interventions and appropriate treatment selection [7, 8]. This systemic approach was mostly investigated in surgical, oncologic patients [9] but not among patients with biliary diseases apart from one study by Lasithiotakis et al. [10]. Furthermore, the appropriate assessment can be even more complex in case of patients with acute cholecystitis. At present, such “patient-related” treatment guidelines do not exist, taking into consideration local pathological changes, patient clinical status and the subsequent treatment decisions [11]. Therefore, the aim of this prospective study was to evaluate postoperative outcomes in frail older patients eligible for elective and emergency cholecystectomy. To our knowledge, this is the first prospective study to assess the outcome of frail older patients undergoing emergency cholecystectomy.

## Methods

### Study population

Between June 2014 and December 2015, we prospectively enrolled consecutive patients over 65 years of age with symptomatic gallstone disease, acute cholecystitis requiring elective or emergency surgery, respectively. The study was conducted at a tertiary referral hospital. The ethics committee approved this study and informed consent was obtained from all patients or their caregivers.

Inclusion criteria for the elective patients were symptomatic and sonographically detected cholelithiasis. Inclusion criteria for the emergency patients were acute cholecystitis according to the 2013 Tokyo Guidelines [12]. Patients with pancreatitis at the time of surgery were excluded. Three patients who were unable to give consent or answer the GA questions were excluded and two additional patients were excluded because of incomplete data.

### Study protocol

All patients were assessed using the GA, which was performed on the day of admission by trained physicians or, for emergency patients, by trained physicians or nurses. The GA comprised the validated instruments presented in the Table 1, with the range and the literature-based cut-off scores [13–20].

**Table 1:** Glossary of the tests used in the geriatric assessment with the range and the literature-based cut-off scores

TEST		NUMBER OF ITEMS	RANGE	CUT-OFF SCORE
ADL [13]	Functional status	6	0–6	<5
IADL [14]		8	0–8	≤7
BOMC Test [15]	Cognitive assessment	6	0–28	>10
CDT-test [16]		7	0–7	>3
Charlson Comorbidity Scale [17]	Comorbidity	19	0–37	>3
Geriatric Depression Scale [18]	Depression	15	0–15	>5
MNA full [19]	Nutritional assessment	18	0–30	<24
Polypharmacy [20]	Polypharmacy	1	0-∞	≥5 drugs/day

*ADL* Activities of daily living, *IADL* Instrumental Activities of Daily Living, *BOMC* Blessed Orientation-Memory-Concentration Test, *CDT* Clock Drawing Test, *CCS* Charlson Comorbidity Scale, *GDS* Geriatric Depression Scale, *MNA* Mini Nutritional Assessment

The results of each test were recorded and each test was also scored on a dichotomous scale, based on whether an impairment in any of the parameters was present.

All operations were performed by residents under the direct supervision of a consultant (who also served as the first assistant) or by the consultants themselves. Laparoscopic cholecystectomy was performed using a standard three- or four-port technique and all emergency patients were treated surgically within 24 h after admission. Severity grading for the acute cholecystitis patients was according to the 2013 Tokyo Guidelines [12].

### Frailty model

A cumulative deficit model of frailty was used. The equally weighted deficits, as a measure of accumulated vulnerability, included ADL/IADL, Geriatric Depression Score, BOMC/CDT, the Mini-Nutritional Assessment, CCS, and the Polypharmacy Assessment. The functional (ADL/IADL) and cognitive domains (BOMC/CDT) were considered abnormal if one of the assessment tools showed literature-based impairment. The detection of deficits in two or more GA domains indicated an increased risk of disability or death and was used as the cut-off score for the GA set and also as the definition of frailty.

### Outcome measures

The primary outcome, complications, was defined as any event occurring within 30 days of surgery that required treatment not routinely applied in the post-operative period. The severity of predefined complications was classified according to the Clavien–Dindo scale [21]. Grade I and II complications were classified as minor morbidity, and Grade III and IV complications as major morbidity. Postoperative mortality was defined as death within 30 days after surgery. LOS was calculated as the period from the admission day until discharge from hospital.

### Statistical analysis

The data were analyzed using Statistica 10.0 software (StatSoft, Krakow, Poland). Categorical variables are described as percentages of the total population, while continuous variables are reported as the median and range. Pearson’s chi-square or Fisher’s exact test was used to compare categorical variables and the unpaired Student’s t-test was used for comparisons.

Univariate and multivariate analysis was conducted to investigate the association between the GA set and 30-day postoperative morbidity (including a comparison of “any” with “no” complications and “major” with “no/minor” complications), adjusted for age and sex. Because of the small number of fatal events, postoperative mortality was not analyzed separately but as part of the “major morbidity” category. Statistical significance was defined as two-sided *p* ≤ 0.05.

## Results

The study sample comprised 66 elective patients (48 female and 18 male) and 60 emergency-admitted patients (34 female and 26 male). The median age was significantly lower among elective patients: 71 vs. 76 years of age; *p* < 0.01. Patients’ baseline characteristics are shown in Table 2.

**Table 2:** Patients’ baseline characteristics

Factor	Elective patients	Emergency patients
Number (female/male ratio) *[n]*	66 (48/18)	60 (34/26)
• 65–74 / 75–84 / 85+ *[n]*	40/20/6	25/27/8
Median age *[years (range)]*	71 (65–86)	76 (65–93)
ECOG-PS [44] *[n]*:		
• 0	19	5
• 1	34	24
• 2	12	12
• 3	1	16
• 4	0	3
ASA [45] *[n]*:		
• 1	8	1
• 2	48	25
• 3	10	30
• 4	0	4

*ECOG-PS* Eastern Cooperative Oncology Group performance status, *ASA* American Society of Anesthesiologists

Laparoscopic cholecystectomy was successfully performed in 57 (86 %) elective and 42 (70 %) emergency patients. The conversion to open cholecystectomy was performed in two patients (3 %) in the elective group and in five patients (8.3 %) in the emergency group. Seven (10.6 %) and 13 (21.7 %) patients had primary open cholecystectomy in the elective and emergency group, respectively, due to severe pulmonary and/or cardiac comorbidities after consultation with the anesthesiologists, numerous laparotomies in the upper abdomen. The mean operation time was 79 min (range, 30–180 min). Emergency frail patients had a non-significantly longer operation time compared with emergency fit patients (85 vs. 71 min; *p* = 0.14). There was no difference between frail and fit patients operated electively (77 vs. 76 min; *p* = 0.87).

The severity of acute cholecystitis in emergency admitted patients according to the revised 2013 Tokyo Guidelines was as follows: Grade I: two patients (3.3 %); Grade II: 39 patients (65 %); and Grade III: 19 patients (31.7 %).

### Descriptive analysis of the GA components and frailty frequency

The Table 3 presents the results of the GA instruments with literature-based cut-off scores and the proportion of patients who had abnormal results in the test.

**Table 3:** Elements of the Geriatric Assessment with the proportion of patients with abnormal test results

TEST	ELECTIVE PATIENTS *[n] (%)*	EMERGENCY PATIENTS *[n] (%)*
ADL (cut-off score <5)		
• Dependent	0 (0 %)	10 (17 %)
IADL (cut-off score ≤7)		
• Dependent	2 (3 %)	19 (32 %)
MNA full (cut-off score <24)		
• Malnutrition	10 (15 %)	24 (40 %)
BOMC (cut-off score >10)		
• Impaired	4 (6 %)	19 (32 %)
CDT-test (cut-off score >3)		
• Impaired	25 (38 %)	25 (42 %)
GDS (cut-off score >5)		
• Depressed	13 (20 %)	19 (32 %)
CCS (cut-off score ≥3)		
• ≥3	9 (14 %)	27 (45 %)
Polypharmacy		
• >5 drugs/day	21 (32 %)	29 (48 %)

*ADL* Activities of Daily Living, *IADL* Instrumental Activities of Daily Living, *MNA* Mini Nutritional Assessment, *BOMC* Blessed Orientation-Memory-Concentration, *CDT-test* Test, Clock Drawing Test ; *CCS* Charlson Comorbidity Scale, *GDS* Geriatric Depression Scale, *p* > 5 Polypharmacy >5 drugs/day

Table 4 shows the results of the cumulative deficit model of the GA. The frequency of frailty was significantly higher among emergency patients compared with elective patients (34 vs. 46 patients, respectively; *p* < 0.01). The cumulative number of abnormal domains among frail patients was between two and five (elective patients) and between two and six (emergency patients). Additionally, significantly more frail emergency patients had Grade III (severe) acute cholecystitis according to the 2013 Tokyo Guidelines (17 vs. three patients, (frail emergency patients vs. fit emergency patients, respectively); *p* < 0.01).

**Table 4:** Validated assessment tools used in the Geriatric Assessment, with frailty frequency

GERIATRIC ASSESSMENT	ELECTIVE PATIENTS	EMERGENCY PATIENTS
ADL/IADL + MNA + BOMC/CDT + CCS + GDS + Polypharmacy (>5 drugs/day)	34 (51.5 %)	46 (76.7 %)

*ADL* Activities of daily living, *IADL* Instrumental Activities of Daily Living, *MNA* Mini Nutritional Assessment, *BOMC* Blessed Orientation-Memory-Concentration, Test, *CDT* Clock Drawing Test, *CCS* Charlson Comorbidity Scale, *GDS* Geriatric Depression Scale

### Outcomes

The discharge status was to home in all elective cases and to skilled nursing facilities in 8.3 % (*n* = 5) of emergency patients. There were no readmissions and there was no mortality among the elective patients. In the emergency group, the 30-day mortality was 5 % (*n* = 3) with patients dying on the 1st, 3rd, and 10th postoperative day because of progressive circulatory insufficiency. The 30-day morbidity was 10.6 % (including 6.1 % major morbidities) and 36.7 % (including 10 % major morbidities), in the elective vs. the emergency group of patients, respectively. Comparing the incidence of both 30-day postoperative overall and major complications between frail and non-frail patients, there was no statistically significant difference (6 % vs. 5 %, *p* = 0.76) in the elective group. All four occurrences of major complications (postoperative bleeding, bile leakage, abscess formation) in the elective group were observed among patients with chronic severe inflammatory processes and were connected to the surgical technique. In comparison, in the emergency group, almost all mortality and major complications were observed in the frail populations (36.7 vs. 3.3 %; *p* < 0.01) - Table 5.

**Table 5:** Severity of all postoperative complications according to the Clavien–Dindo classification among frail and non-frail elective and emergency patients

Clavien-Dindo classification	Number of complications			
	Elective patients		Emergency patients	
	Frail	Non-frail	Frail	Non-frail
Grade I	1	1	1	1
Grade II	2	1	10	2
Grade IIIa	0	0	1	0
Grade IIIb	2	2	2	0
Grade IVa	0	0	5	0
Grade IVb	0	0	0	0
Grade V	0	0	3	0

Comparing the LOS of frail and non-frail patients, the frail group had non-significantly longer mean hospital stay (5.6 vs. 4 days; *p* = 0.22) in the elective group. In the emergency group, the LOS was significantly longer among frail patients (10.3 vs. 6 days, frail emergency patients vs. non-frail emergency patients, respectively; *p* = 0.03).

### Cumulative effect of GA in determining surgical outcome

The results of the univariate and multivariate logistic regression analysis showed that age and sex did not increase the surgical risk either in the elective or the emergency group. The frailty status was not predictive of all and major postoperative complications in the elective group. In turn, frailty was an independent risk factor for all complications in the emergency group. It was not possible to build a model for the major complications in the emergency group because of an insufficient number of patients in this subgroup (major complications were observed only among frail patients). The results of the multivariate logistic regression are presented in Table 6.

**Table 6:** Results of the multivariate logistic regression of the cumulative number of impairments in the Geriatric Assessment set on postoperative outcome (adjusted by age, sex)

GERIATRIC ASSESSMENT	ELECTIVE PATIENTS OR (95 % CI)		EMERGENCY PATIENTS OR (95 % CI)	
	ALL	MAJOR	ALL	MAJOR
ADL/IADL + MNA + BOMC/CDT + CCS + GDS + Polypharmacy (>5 drugs/day)	1.2 (0.5-2.7) *p* = 0.63	0.89 (0.3-2.6) *p* = 0.84	3.4 (1.2-9.7) *p* = 0.02	-^a^

All variables were considered dichotomous; *OR* odds ratio, *95% CI* 95% confidence interval, The statistically significant values appear in bold font; ^a^There were no cases of major complications in the non-frail group. *ADL* Activities of Daily Living, *IADL* Instrumental Activities of Daily Living, *MNA* Mini Nutritional Assessment, *BOMC* Blessed Orientation-Memory-Concentration ,Test, *CDT* Clock Drawing Test, *CCS* Charlson Comorbidity Scale, *GDS* Geriatric Depression Scale

## Discussion

In this study, almost half of the elective and two thirds of the emergency older patients were frail. However, the procedure was performed safely with no 30-day postoperative mortality and a low number of major complications, for both frail and non-frail elective patients. In contrast, we encountered poorer outcomes in emergency operated patients. All of the cases of mortality and major comorbidity occurred in frail emergency patients, which was confirmed by regression analysis. Frail status was not a risk factor for overall and major postoperative complications in the elective group but was a significant independent predictive factor for postoperative complications in the emergency population. Similarly, the LOS was not significantly longer in the elective frail group of patients and was significantly longer in the emergency frail population.

Published studies show that elective laparoscopic cholecystectomy is a feasible and safe procedure in older patients, including in a group of octogenarians. The authors report no or single cases of mortality and usually a higher, but acceptable, rate of postoperative complications when compared with a younger population [1–6, 22, 23]. However, the authors of a meta-analysis of laparoscopic versus open cholecystectomy in older patients, including studies up to June 2013, concluded that further high-quality evidence is necessary to draw definitive conclusions, although the best available evidence supports the selective use of laparoscopy [24]. Older patients with acute cholecystitis have more frequent primary open cholecystectomies, a higher conversion rate and, in most studies, a worse postoperative outcome [25–28]. We obtained similar results with no mortality in the elective population, a low postoperative complication rate, and most of the procedures were performed using minimally invasive techniques. Similar to other studies, older patients with acute cholecystitis had a higher rate of mortality, morbidity, primary open cholecystectomies, and conversions in our study. However, the frailty factor was not considered in these studies and advanced age alone is not necessarily synonymous with vulnerability to adverse health outcomes.

Another problem with the frailty concept is the choice of the best assessment model, which is still debatable. Three of them are most frequently cited in the current literature: the multi-domain GA, the cumulative deficit score (CDS), and the frailty phenotype model [7]. Most trials evaluating the multi-domain GA in surgical patients have analyzed the relationship between individual elements of the GA and the outcomes, showing a statistically significant predictive possibility of postoperative complications [29–32]. In our study, domains as a single risk factor were not relevant in the elective patients, both in the univariate and multivariate regression analysis. In turn, in the emergency group some domains (I-ADL, CDT, CCI, Polypharmacy) turned out to be the risk factor of postoperative outcome, however, only in the univariate analysis (data not included in the paper).

Robinson et al. and Kim KI et al. showed that a cumulative deficit frailty score was independently associated with postoperative mortality [33, 34]. Similarly, in our previous study, this model turned out to be also predictive of 30-day postoperative morbidity in oncologic patients with solid cancer eligible for abdominal surgery. However, the number of incorporated GA domains had a great influence on the prevalence of frailty and on adequate surgical risk assessment [35]. Another problem is the cut-off of two abnormal domains, which was used as a frailty definition in our study. We extrapolated the guidelines of Geriatric Assessment used by the SIOG in the older oncologic patients [36]. However, there is still no “golden standard” regarding the number of impaired domains that should be considered as a frailty marker.

Kristjansson et al., Kwang-il et al., Tan et al., and Makary et al. used a frailty phenotype model and observed that impairments were also independently associated with adverse in-hospital events, prolonged LOS, and post-discharge institutionalization [37–40].

However, none of these studies analyzed exclusively patients eligible for cholecystectomy. Lasithiotakis et al. performed the only study including a frailty assessment in 57 older patients eligible for elective laparoscopic cholecystectomy. In contrast to our results, they observed that the GA might predict 30-day postoperative complications and a prolonged stay. This and our study have common features: similar median age, a cumulative model used to assess frailty (with five out of six similar domains and the same cut-offs), almost the same number of patients identified as frail (56.1 vs. 51.5 %), and a similar method of reporting outcomes [10]. However, our study has a few important differences. The study by Lasithiotakis et al. included only patients with uncomplicated biliary disease whereas our study included consecutive patients also presenting with severe chronic cholecystitis (intraoperative finding in patients admitted electively) and these patients experienced the major postoperative complications. Unfortunately, our study period included the highest number of bile leakages ever experienced in our institution, in both the fit and frail groups. Moreover, our study included also the Geriatric Depression Scale in the frailty assessment, which is a proven predictor of postoperative adverse outcome [41].

Although, we are in favor of the use of the cumulative deficit model, it still remains unclear which measure of frailty should be used in surgical patients. All of the frailty models evaluate the same concept but from a different perspective and further research is needed to determine the best approach.

It is also important to note the severity of acute cholecystitis in our case series. Previous studies have shown that perioperative outcomes are influenced by the severity of gallbladder disease rather than chronological age [42]. In our emergency population, only 3 % of patients had a Grade I score and more than one third had Grade III acute cholecystitis according to the 2013 Tokyo Guidelines, which is one of the highest reported results. Moreover, the timing of the surgery plays an important role [43] but this factor was not significant in regression and correlation analysis (data not presented) in our study.

We believe that the severity of the surgical insult is of great importance in frail patients. In our study, in patients eligible for elective cholecystectomy, this factor did not appear to be strong enough to influence the reduced physiologic reserve and outcomes in frail patients and this could be a result of further heterogeneity within this population. The identification of frailty alone appears to be insufficient in patients undergoing surgery and it should be supported by its magnitude. The number of abnormal deficits in the cumulative model of frailty could be one such factor. Additionally, meticulous surgical technique in experienced hands is of paramount importance for this particular group. Therefore, the influence of the surgeon, the surgical technique, and the reaction of the patient’s body to the stress connected with the operative procedure in the context of frailty require further study.

## Conclusion

Elective laparoscopic cholecystectomy is a safe and effective surgical technique also for older frail patients. In emergency settings, frail patients have significantly more complications and a longer LOS compared with the fit population. However, the role of severity of frailty and the most reliable GA tools require further study.

## Abbreviations

ADL, activities of daily living; ASA score, the American Society of Anesthesiologist’s score; BOMC, the blessed orientation-memory-concentration test; CCS, the charlson comorbidity scale; CDS, the cumulative deficit score; CDT, the clock drawing test (CDT); ECOG-PS, the performance status eastern cooperative oncology group performance status; GA, geriatric assessment; GDS, the geriatric depression scale; IADL, instrumental activities of daily living; LOS, length of hospital stay; MNA, the mini nutritional assessment
